# Dependent, Poorer, and More Care-Demanding? An Analysis of the Relationship between Being Dependent, Household Income, and Formal and Informal Care Use in Spain

**DOI:** 10.3390/ijerph18084339

**Published:** 2021-04-19

**Authors:** Beatriz Rodríguez-Sánchez, Marta Pascual Sáez, David Cantarero-Prieto

**Affiliations:** 1 Faculty of Communication and Humanities, University Camilo José Cela, Villanueva de la Cañada, 28692 Madrid, Spain; 2 Research Group of Health Economics and Health Services Management IDIVAL, Department of Economics and GEN, University of Cantabria, Avenue Los Castros, s/n, 39005 Santander, Spain; marta.pascual@unican.es (M.P.S.); david.cantarero@unican.es (D.C.-P.)

**Keywords:** limitation, dependency law, ageing, household income, long-term care, income-related gradient

## Abstract

Population ageing is one of the current challenges that most societies are facing, with great implications for health systems and social services, including long-term care. This increasing long-term care use is particularly rising for dependent older people, motivating the implementation of regional dependency laws to ensure their care needs’ coverage. Using data from the Survey of Health, Ageing, and Retirement in Europe (SHARE) from the year 2004 until 2017, the aim of this study is to assess the impact that the Spanish System for Personal Autonomy and Dependency might have on (i) household income, according to different needs for care levels, by running Generalized Linear Models (GLMs); and (ii) formal and informal care use depending on the income-related determinant through the performance of logit random-effects regression models. We show that the different degrees of needs for personal care are associated with a lower household income, being associated with an income reduction from €3300 to nearly €3800, depending on the covariates included, per year for the more severely in-need-for-care older adults. Moreover, our findings point towards a higher use of formal and informal care services by the moderate and severe dependents groups, regardless of the household income group and time period. Bearing in mind the demographic ageing, our results highlight the need for the identification of potentially vulnerable populations and the efficient planification of long-term care systems and social support services.

## 1. Background

### 1.1. Introduction

Many countries are facing a growth in the number and proportion of older people in their populations, which is likely to have implications for the health and social protection systems [1,2]. Moreover, such demographic changes might also imply an increase in the share of the population presenting lower functional capacity, requiring assistance for daily living activities and a greater demand for Long-Term Care (LTC) in the coming years [3]. LTC expenditure represents 1.4% of the Organization for Economic Co-Operation and Development (OECD) countries’ Gross Domestic Product (GDP) in 2014 [4,5,6], with large differences between countries, ranging from 4% in The Netherlands to less than 0.5% of GDP in some other countries such as Israel, Latvia, and Poland. However, this figure was estimated to more than double by 2050 [7]. The expected growth in LTC expenditures as a share of GDP and of public and private spending can be explained by population ageing [8,9], the greater probability of survival to older age [10], and the decline in the supply of informal caregiving due to some major social changes (i.e., new family structures, lower household size, higher female labor market participation) [9,11].

Hence, the aim of this study is to assess the impact that the Spanish System for Personal Autonomy and Dependency might have on household income and formal and informal care use.

### 1.2. Background Knowledge

The interrelationship between the different components of long-term care (mainly formal and informal care) is widely studied in the literature. Traditionally, informal care was regarded as a substitute of nursing homes [12,13]. Actually, a study using data from the Survey of Health, Ageing, and Retirement in Europe (SHARE) showed that informal and formal care are substitutes as long as the elderly’s disability is low [14]. Substitutability or complementarity between informal care and formal care outside the household were largely discussed, highlighting that differences can be found regarding the disease, the services provided, and the degree of disability of the care recipient [13,15,16,17,18]. Furthermore, variation within use of informal care services is quite large within European countries, not only due to population distribution and population ageing, but also due to the design of welfare programs in Europe, and the availability of support to these caregivers. For example, in Mediterranean countries, as Spain is, where informal care tradition is common, the benefits and support that informal caregivers receive for their services are quite low. On the other hand, in Northern European countries, informal care is not so extended, but social benefits and support are higher. Finally, in Central Europe, caregivers are provided with widely spread social support programs, benefits that vary within and across regions, but informal care is not so relevant [19,20].

Furthermore, differences in LTC demand can be explained by household characteristics, such as household composition and income, while playing a more modest role is the education and geographical location [21], as supported by the literature. However, other determinants of LTC might be limitation thresholds and how much coverage of care need is to be a public or a private responsibility [10], leading to economic and social inequalities [10,22]. In another study, the authors found different relationships between the type of long-term care service received and household income. More precisely, the authors found that the sole use of informal care was reduced with higher household income, whereas receiving both types of care, formal and informal, was associated with higher household income [18]. On the other hand, the sole use of formal care increased among the poorest households.

Moreover, international differences in LTC were studied from different points of view. Bakx et al., (2015) [23] concluded that LTC use is affected by country-specific eligibility criteria for public LTC coverage and comprehensiveness of public LTC systems. In the case of Spain, by the end of 2006, a new System for Promotion of Personal Autonomy and Assistance for Persons in a Situation of Dependency (SAAD) was released through the approval of Act 39/2006 on 14 December [24]. This Dependency Act (DA) recognized the universal entitlement of Spanish citizens to social care services according to their limitation degree, entering as the fourth pillar of Spain’s Welfare State [25], in addition to health, education, and pensions. In Spain, the support and LTC to older people in need for personal care was traditionally organized within the family, mainly provided by women, being sometimes complemented by formal care [26,27]. Hence, one of the purposes of the DA was to reduce the burden of family members who undertake the role of the primary caregiver, and who additionally benefited from being registered within the Social Security System, recording their employment status as non-professional carers. Furthermore, the new system aimed to guarantee an adequate number of resources and services (i.e., prevention and promotion of personal autonomy, home help, day and residential care) to satisfy the growing demand and use of long-term care due to population ageing [28]. Still, public bodies were limited to provide LTC services only in cases where household income was not enough to cover such needs and if the older adult in need for care had a high grade of functional limitations [29].

Three levels of functional limitation were defined by the DA (mild, moderate, severe) with older adults in need for personal care, classified according to an official scale [30,31], which consisted of 47 tasks later grouped into ten activities of daily living (feeding, control of physical needs, toileting, other physical care, dressing, maintaining one’s health, mobility, moving inside and outside the household, and being able to do housework). According to the score obtained in those 47 domains, the severity of the functional limitations was classified as: not eligible (0–24 points); mild level 1 (25–39 points) and level 2 (40–49 points); moderate level 1 (50–64 points) and level 2 (65–74 points); and severe level 1 (75–89 points) and level 2 (90–100 points).

At the end of the year 2013, 1,644,284 applications were received. From these, around 60% (944,345 requests) were eligible, but only 753,842 were actually receiving their benefits by December 2013 [32]. Moreover, despite the fact that SAAD was designed to provide universal coverage to older people in need of personal care, when the SAAD was fully active in 2015, 33.7% of the financial contributions were supported through co-payments afforded by the individuals who benefited from the DA. Moreover, according to an assessment of the Act, 45.5% of the finally perceived benefits were economic (cash-for-care) for the informal care provided by any family member who acted as the main caregiver [33], being much more extensively employed than planned. Another issue that should be considered is the fact that the 2008 economic crisis added more uncertainty to the system process, mainly due to inequality in access to LTC services between regions [34].

### 1.3. Purpose

To the best of our knowledge, there is no study in the existing literature that aims to assess the influence, through the application of appropriate statistical approaches, of the DA on household income and the use of formal and informal care, by additionally evaluating the mediation effect that income might have had on such care reception. Hence, the aim of this study is to assess the impact that the Spanish System for Personal Autonomy and Dependency might have on some outcomes depending on the income-related determinant, according to several characteristics of the Spanish population. Our purpose is, then, two-fold: (i) we aim to assess the association between being in need of personal care and household income, and (ii) the relationship between the different functional limitations and income levels on the use of formal and informal care. The hypotheses we aim to asses are (i) that the implementation of the DA would have had a positive impact on household income, since one of the benefits considered in the law was to receive cash benefits for those individuals who received non-professional care; and (ii) there were income inequalities according to the income level, which were also dependent on the functional status of the older individuals.

## 2. Materials and Methods

### 2.1. Sample Data

The data used comes from the Survey of Health, Ageing, and Retirement in Europe (SHARE). SHARE emerged as a longitudinal survey with information on more than 120,000 individuals aged 50 years old and above from 27 European countries plus Israel. For ease of understanding of the data used in the study, more information can be found in Börsch-Supan et al., (2013) [35]. The period of analysis will cover 2004 (wave 1), 2006/2007 (wave 2), 2010 (wave 4), 2013 (wave 5), 2015 (wave 6), and wave 7 (2017). Wave 3 is not included due to a change in the SHARELIFE questionnaire and, hence, the information provided in Wave 3 was not useful for our analysis.

Given the aim of the study, we selected the individuals who reported to be living in Spain at the date of the interview with a minimum follow-up of three waves, which should be: the time before the DA (wave 1, year 2004), in the year of the introduction (wave 2, year 2006/2007), and after the DA (wave 4, year 2010; wave 5, year 2013; wave 6, year 2015; or wave 7, year 2017). Hence, after selecting the observations with information on at least three waves (two of them being wave 1 and 2, and then, at least, wave 4, 5, 6, or 7), and the individuals with non-missing values in any of the variables considered in our analysis, our sample further decreased to 4364 observations.

### 2.2. Selection of Variables

#### 2.2.1. Dependent Variables

Two dependent groups of variables comprise the outcomes of the current study: The first outcome refers to household income, which is a continuous and self-reported variable referring to all annual income that was received by all the members within the same household. Household income is calculated as the sum of individual income from the responder (which is obtained from the individual income from employment, self-employment, pension, private regular transfers (i.e., alimony), and long-term care), as well as from the sum of the gross incomes of other household members and other benefits, capital assets income (income from bank accounts, from bonds, from stocks or shares, and from mutual funds), and the rent payments received, plus imputed rents.

The second group of outcomes is formal care and informal care use. In case of the former outcome, information will be taken on whether the individual received professional help at home, as well as nursing home use, either permanent or temporarily, in the previous 12 months. About professional help at home, the questionnaire contains information on whether the individual received professional help at home with various matters, such as meals on wheels or cooking. However, it should be noted that the question related to home care was excluded in the questionnaire of Wave 4. Hence, the only measure of formal care available in Wave 4 is nursing home care as “institutions sheltering older persons who need assistance in activities of daily living, in an environment where they can receive nursing care, for short or long stays”. Thus, the dependent variable took a value 1 if the respondent made use of any of the professional services mentioned above, and 0 otherwise. For informal care, SHARE allows for the identification of whether a non-professional caregiver, from inside or outside the household, helped the survey respondent due to any limitation in the activities of daily living during the previous 12 months.

#### 2.2.2. Independent Variables

##### Being Identified as in Need for Personal Care According to the Dependency Act

As Table S1, Supplementary Material, shows, the definition of “dependency” as an older adult in need for personal care in the Dependency Act was based on the limitations in the basic and the instrumental activities of daily living. SHARE does contain responses to the Katz Activities of Daily Living (ADL) Index [36,37]. This index, usually referred to as the Katz ADL, evaluates functional status as a measurement of the person’s ability to carry out six activities of daily living independently. These are bathing, dressing, toileting, transferring, continence, and feeding. Moreover, SHARE also includes information on the number of limitations in the Instrumental Activities of Daily Living (IADL). This scale, usually referred to as the Lawton’s IADL scale, evaluates the individual´s ability to perform eight instrumental activities of daily living [38]: telephone use, shopping, cooking, housekeeping, laundry, transportation, preparation of own medication, and financing. Considering the weight assigned to it and the different categories within each, we generated our dependency score, given the availability of questions in SHARE.

According to the score obtained following the weights and points in Table S1, which are derived from the Dependency Act classification according to the individual´s limitations in both ADLs and iADLs, the severity of the functional limitation was classified as: not eligible (0–24 points), mild (25–49 points), moderate (50–74 points), and severe (75–100 points).

##### Household Income Groups

In the second-group analysis, whose outcomes are formal and informal care reception, household income was divided into tertials, according to the distribution of the original household income variable, which was previously described as: low household income (income ranging from €0 to €14,135.19, annually), medium household income (€14,138.43–€29,046.13 per year), and high household income (€29,088.67 per year as the minimum and €477,483.8 as the maximum)

##### Other Independent Variables

There are three types of individual determinants of health and social care use: predisposing factors, which determine the individual’s predisposition towards the use of resources, in this case long-term care; the enabling factors, which refer to the resources available to satisfy a health need; and need factors, which require the reason why the individual, due to the above factors, requests health care [39].

With respect to health status, which would enter as a need factor, different variables entered the analysis: self-assessed health status [40,41], number of chronic conditions (denoting the sum of the following conditions: heart attack, high blood pressure or hypertension, high blood cholesterol, a stroke or cerebrovascular disease, diabetes or high blood sugar, chronic lung disease, cancer or malignant tumour, stomach or duodenal or peptic ulcer, Parkinson disease, cataracts, and hip or femoral fracture), and a dummy variable for depression.

Moreover, other variables are the predisposing factors towards the use of formal and/or informal care. These were age, gender, level of education (no education, low, medium, and high, according to ISCED-97 codes), marital and employment status, number of children and grandchildren, whether any children lived in the household, and body mass index categories. Lastly, and only in case of the second aim of analysis, formal and informal care use, as enabling factors, entered the analysis as appropriate, depending on the outcome assessed.

### 2.3. Statistical Analyses

#### 2.3.1. Analyzing Associations between Being in Need for Personal Care and Household Income

The marginal impact of being functionally limited and the starting time of the Dependency Act, as well as the other independent variables on household income, were estimated using a Generalized Linear Model (GLM), given the skewed distribution of income [42]. GLMs are empirical transformations of the classical ordinary least square (OLS) regression model, which specify the conditional mean function directly. Specifically, GLMs do not require transformation scales, but a response distribution of one of the exponential family of distributions, which relates the mean of the response to a scale on which the model effects combine additively [43]. According to the Modified Park Test, the chosen family was the Gamma distribution for modeling household income in our analysis, with an identity link.

We ran four regressions models. In Model 1, we included wave dummies, the different categories of functional limitation level, and the interaction between those two categorical variables, as well as age, gender, education, and marital status. Then, in a second regression model, we included employment status. To Model 2, the third regression model added living conditions, such as living in a rural area or the number of children and grandchildren. Finally, health status variables were introduced in a fourth regression model.

#### 2.3.2. Assessing the Impact of the Different Levels of Limitations and Household Income on the Reception of Formal and Informal Care

Modelling the probability of a positive outcome with a linear probability model (LPM) is a problematic issue. Instead, non-linear models for binary responses such as logit regressions with random effects were estimated [44,45,46]. We clustered standard errors at the individual level with the aim to correct for the existing correlation between individuals’ different observations across waves.

In logit models, estimated coefficients capture the effects on the log-odds-ratio (see e.g., Heij, C. et al., 2004 [44]). Let $\Lambda \left(t\right)=e^{t}/\left(1+e^{t}\right)$ be the logistic function with values stretching between zero and one, and let

(1) $$Pr[formal_{it}=1\;|x_{it}]=\Lambda \;\left(x_{it}^{\prime }\beta \right)$$

where *i* represents the individual, and *t* wave. $formal_{it}$ is a dummy variable indicating that respondent *i* received formal care in year *t*. $x_{it}^{\prime }\;$ is a vector of explanatory variables, which, in Model 1, includes time dummies, functional limitation levels, the different household income categories (low, medium, and high), as well as sociodemographic indicators (age, gender, marital status, and education level) and a dummy variable in case respondent *i* receives informal care in time *t*.

Model 2 adds employment status to Model 1. In Model 3, we additionally control for living conditions, which would consist of living in a rural area, the number of children or grandchildren, and whether any of these children live within the household. In Model 4, variables related to the health status (self-assessed health status, number of chronic conditions, and depression) and the healthy lifestyles (body mass index categories) are added.

The same procedure is followed for our second outcome of interest, to receive informal care, either within or outside the household. It should be noted that, when informal care was the outcome of interest, a dummy variable for formal care reception entered the regression instead

A coefficient is assumed to be significant when it is statistically significant, at least at 5% (95% confidence level).

## 3. Results

### 3.1. Summary Statistics

Table 1 shows the summary statistics of the sample for the set of covariates included in the analysis by year. Descriptive variables were compared between waves through T-tests or Chi-square tests for continuous and categorical variables, respectively.

Table 1 shows some differences in the sociodemographic characteristics and living conditions of the individuals. Mean household income decreases from €24,330, approximately, in 2004 to €15,238 in 2015, when the household income reached its minimum. 

The proportion of people receiving formal care was lower in 2006/2007, when the DA was announced, than in the year 2004, but the proportion of formal care receivers increased in the following years after the implementation of the DA. However, the increase in the use of formal care services between years seems to be driven by the demand for homecare rather than nursing home care. On the other hand, the proportion of people receiving informal care (inside or outside the household) increased between years but decreased in the last year included in the analysis. The same trend was followed for both types of informal care, inside and outside the household. 

With respect to the functional and health status, individuals seem to be less healthy in later years than at the beginning of SHARE, as the proportion of people being classified as “severe functional limitation” increased (from 0.21 in the year 2004, on average, to 3.64 in 2017), as well as the percentage of individuals within the category “no limitation” decreased from 97.02% to 89.91%.

### 3.2. Regression Results

#### 3.2.1. Analyzing Associations between Being in Need for Personal Care and Household Income

The results from the GLM regression with Gamma distribution and identity link on household income (Table 2) show that, in the baseline model (Model 1), functional limitation levels were not significantly associated with household income. Compared to the reference category (wave 2, years 2006/2007), living in wave 1 (year 2004) was significantly related to higher household income, which increased by €2686. None of the interactions between the limitation levels and the waves emerged as significant predictors. In Model 2, when employment status categories were considered, the set of limitations categories and their interaction with time dummies were not significant at 5% either. The coefficient from wave 1 was still significant and increased compared to Model 1, being €2857. When living conditions were included (Model 3), compared to being non-limited, severe need for personal care was significantly related to lower household income, which decreased by €3314. Living in wave 1, compared to wave 2 (years 2006/2007), was associated with higher household income, which became higher by €2275. As in models 1 and 2, none of the interactions was significant either. In Model 4, health variables entered the analysis. Being severely in need for personal care decreased household income by €3771, compared to no limitation. Being in wave 1 (year 2004) was still significantly related to higher household income, but with a lower coefficient (€2298). Interactions were not significant at 5%.

#### 3.2.2. Assessing the Impact of the Different Levels of Functional Limitation and Household Income on the Reception of Formal and Informal Care

Table 3 shows the results from the logit regressions performed on formal care use. Compared to no limitation, all the functional limitations levels were significant and positively related to formal care reception. For example, being moderately in need for personal care was significantly associated with formal care, which was 7.11 OR higher than for people with no limitation. In the case of severe functional limitations, the odds ratio dropped to 4.02. Compared to wave 2 (years 2006/2007), living in wave 4 (year 2010, the first wave after the implementation of the DA at the end of 2006) was significantly related to formal care reception. Household income individually nor jointly with functional limitations levels was significantly associated with formal care reception. The odds ratio from the different levels of limitation decreased in Model 2, when employment status was included, compared to Model 1, but still significant. Moderate needs for personal care was significantly related to the odds of receiving formal care by 5.57 OR. Severe functional limitation was no longer significantly associated with formal care. Being in wave 4 was also significantly related to formal care reception, but with a lower coefficient than in Model 1. When living conditions (Model 3) and health status (Model 4) were considered, being moderately in need for personal care was significantly associated with the odds of formal care use, increasing its coefficient compared to the previous specifications (OR 9.11 in Model 3 and 9.06 in Model 4). Household income and its interaction with limitation categories were never significant. On the contrary, receiving informal care was always significantly related to a higher probability of formal care use, pointing towards the complementarity between both types of long-term care.

Table 4 displays the results from the logit regressions performed on informal care reception. Compared to non-limited people, all the limitation levels were significant and positively related to informal care use, with the moderate need for personal care level having the greatest odds of using informal care (OR 75.05). Compared to wave 2 (years 2006/2007), living in wave 6 (year 2015) was positively and significantly related to informal care reception (OR 0.55). Although also significant and with a negative relationship, wave 7 reported a lower odds ratio than wave 6. Low household income, compared to high household income, was significantly associated with the odds of informal care use (OR 1.26). One of the interactions between household income and functional limitations emerged as a significant predictor of informal care reception: being moderately in need for personal care and having low household income were significantly related to higher odds of informal care reception (OR 0.11). The odds ratio from the limitation levels became lower in Model 2, when employment status was included, compared to Model 1, but still significant. Moderate limitation was significantly associated with the odds of using informal care by 69.80 OR. Severe functional constraint was significantly related to informal care reception, which increased by 26.48 OR compared with non-limited older adults. Low household income was no longer significant at 5%, nor was the interaction between moderate limitation and low household income. When living conditions (Model 3) and health status (Model 4) were considered, all limitation levels were significantly associated with the probability of informal care, increasing the coefficient from moderate functional limitation in Model 4 compared to the previous specifications (OR 72.37 for moderate need for personal care). Household income categories were not significant, but the interaction between moderate limitation and low household income was still significant (OR 0.083 in Model 4). Wave 6 was still significant and positively related to a higher probability of receiving informal care. With respect to the formal care reception variable, it was significant and positively related to a higher probability of informal care reception across regression models, confirming the complementarity between both kinds of long-term care.

## 4. Discussion

The aim of this research was to analyze the impact that the Spanish System for Personal Autonomy and Dependency might have on (i) household income, and (ii) on the use of formal and informal care, depending on the income-related determinant, according to several characteristics of the Spanish citizens.

Our findings suggest that the different functional limitation degrees are associated with a lower household income only after adjusting for living conditions (Model 3) and health status (Model 4). The results obtained showed that being in need of personal care was associated with an income reduction in household income from €3300 to nearly €3800 per year for older adults that were severely in need of personal care. The rationale behind the result might be that more care-demanding households might incur higher healthcare expenditures [47,48], which might even lead to catastrophic health spending [48]. Our insights would be in line with the conclusions driven by other authors in other European countries, such as Ireland [49] or the United Kingdom [50], who found that household income steeply decreased when living with a disabled person. However, the interaction between the functional limitation level and the time dummies was never significantly related to household income. Furthermore, the only time dummy that emerged as a significant factor associated with household income was the time dummy referring to the period before the DA (wave 1, year 2004), leading to increases in household income.

The mechanism behind the decrease in household income according to the need for personal care level might be explained by several issues: first, the fact that disability and dependency onset might lead to work incapacity for those not yet in the retirement age; second, higher demand and use of informal caregiving, implying that some relatives might reduce their working hours or even retire early; thirdly, increased costs of living due to a higher demand of health and formal paid care, which would increase the associated expenditure, reducing the available income for other goods. Furthermore, the new system aimed to guarantee an adequate amount of resources and services to satisfy the growing demand and use of long-term care [28]. Still, public bodies were limited to provide LTC services only in cases where household income was not enough to cover such needs and if the older adult in need of care had a high grade of functional limitations [29]. However, a recent study showed that only 10% of the informal care time provided by family caregivers was eventually covered by the government [51]. Our findings confirm that the moderate and severe functionally-limited older adults have a higher use of formal and informal care services, regardless of the household income group and the time period. In fact, we observe that moderate needs for personal care are associated with a higher probability of formal and informal care reception, compared to severe limitations, which would be consistent with previous estimates [10,21]. The different functional limitations levels were still significantly associated with both outcomes even after controlling for individual and household characteristics, which were assumed to be related to inequalities in long-term care access [21]. Another relevant result concerns the positive relationships between informal and formal care use, pointing towards a complementarity association between both LTC services, as was already found in the existing literature [13,14]. Still, differences might be present according to the need for personal care of the care receiver [13,18].

Moreover, if coefficients were comparable between formal and informal care, our estimates suggest that the effect of functional limitations is higher on informal than formal care, as the odds of using informal care by an individual with moderate needs for personal care is 72.37 larger than the odds of using such care among people with no dependency. However, although statistically significant, the effect drops to OR 9.06 in the case of formal care reception. The vast impact of informal care among adults in need for personal care was indeed estimated to represent around 1.73–4.90%, depending on the dependency level, of the Spanish GDP [52], which reflects the burden that care for functionally limited people poses on the society. Household income and its interaction with the limitations levels were barely significant across regression models and long-term care services, pointing towards the predictive power of need for personal care itself.

In order to correct for structural changes that might happen simultaneously with the implementation of the SAAD, especially the 2008 economic crisis and consequent budget cuts, we included time dummies for the survey waves, taking as a reference wave 2 (years 2006/2007). It should be noted that the recent financial crisis brought about important drops in health and care services, as long-term care is, in addition to high unemployment rates (which raised 27% in 2014 in Spain) in addition to a higher risk of social exclusion [53]. Furthermore, after the worst crisis times (2009–2013), new regulations led to a substantial reduction in public expenditure and a higher promotion of co-payments. Home help shortcuts, in addition to a relevant delay in the evaluation of benefit applications under the DA, mainly those affecting moderate and mild levels of limitations, led to the existence of the so-called “dependency limbo” for those who were actually entitled to receive the benefits observed by the DA but eventually received none [27].

Some limitations should also be mentioned. First of all, we point out the generation of the different levels of functional limitations. As Table S1 shows (Supplementary Material), SHARE does not include as many activities of daily living as the Dependency Act considered. However, the use of weights and information included in SHARE might have reduced such bias. Secondly, we were not able to analyze the effect in the first wave right after the declaration of the DA, as the data that correspond to 2008 (wave 3) refer to individuals´ childhood conditions. Hence, the first-time reference of observation is 2010, that is, four years after the DA, when the immediate effect might have smoothed. We consider that having three points in time after 2006 provides consistent and trustworthy estimates. Thirdly, and regarding the second part of analysis (limitations and household income on the use of formal and informal care), the results from 2010 (Wave 4) for formal care should be interpreted with caution, as information on home care was excluded in questionnaire of Wave 4. Hence, the only measure of formal care available in wave 4 is nursing home care.

In recent times, there were huge advances in the development of empirical studies of public policy evaluation. This fact is motivated both by the greater availability of data at the microeconomic level (especially from surveys based on microdata) and by the latest computer advances. Actually, despite the availability of large panel datasets, we were not able to assess fully the impact of the DA. The study of the impact of aging and limitations of individuals as well as their redistributive incidence is still an open research field with a significant potential for advancement within the Welfare and Health Economics, taking into account the wide diversity of diseases, many of them based on chronic factors. Hence, it would be desirable to collect detailed information on the eligible individuals and those eventually obtaining the benefits. It is true that the Spanish Ministry of Health had a survey called EDAD on disability, personal autonomy, and dependence, but the last available data are from 2008. Future lines of research could entail collecting data from the actual applicants who were not eligible, the eligible ones, and the people who already received any benefit considered within the law.

Our results suggest that the introduction of the Dependency Act, instead of alleviating the burden assumed by informal caregivers in the care provision, posed an even greater burden, heavily increasing its use, which was not parallel to the increase in formal care availability at all. However, the heavy caring load sustained by the informal caregivers was not accompanied by the cash benefits promised at first within the new law [54], but later significantly decreased. Hence, governments should take into account that although informal care promotion is tempting from a public policy perspective due to its free provision, the heavy burden borne by informal caregivers should not be neglected, as its impact on national expenditures is vast enough [52]. Policymakers should answer to the dramatic situations that informal caregivers might face, especially when they give up their job to satisfy their caregiving tasks, and design the appropriate policies, additionally promoting the use of formal care services. Nevertheless, the successful policies implemented to ensure fair access to affordable social care are complex to assess from a comparative view, and it is even more difficult to determine LTC public policy recommendations applicable to heterogeneous welfare models, so our approach is more suitable for the case of Spain.

The increase in the older population in Europe will continue in the coming years and will pose new challenges in the reorganization of both formal and informal care for functionally impaired older adults, in addition to access to better information on the factors that determine them for a coordination of social services in efficiency and equity.

## 5. Conclusions

This study shows that, although the evolution of chronic limitations in Spain depends on socioeconomic inequalities, there are other important directions for future research related to (i) how being limited in performing activities of daily living affects household, and not only individual, income; and (ii) how being functionally limited and household income are associated with the use of formal and informal care, especially after the introduction of laws that aim to cover the particularities of older adults in need of personal care, such as the Dependency Act in Spain. Higher levels of limitations report large decreases in household income, compared to non-limited individuals, maybe due to higher long-term care needs, as our results also show. Taking into account the ageing demographic context that European societies are facing, our results point out the necessity to identify potentially vulnerable populations and to enhance the efficient planification of long-term care and social support services.

## Figures and Tables

**Table 1:** Descriptive statistics by year in Spain, *n* = 4364.

Variables	Wave 1, Year 2004 (*N* = 971)	Wave 2, Year 2006/07 (*N* = 959)	Wave 4, year 2010 (*N* = 826)	Wave 5, Year 2013 (*N* = 779)	Wave 6, Year 2015 (*N* = 419)	Wave 7, Year 2017 (*N* = 410)	Comparison of Means *p*-Value
*Formal care*	5.92	5.69	0.84	8.89	10.51	8.79	0.000 ***
Nursing home	0.31	0.21	0.84	0.51	0.23	0.43	0.183
Homecare	5.62	5.48	-	8.77	10.28	8.73	0.000 ***
*Informal care*	16.14	18.82	20.50	23.51	18.93	22.83	0.004 ***
Informal care from outside the household	11.95	12.72	15.59	16.90	15.19	17.04	0.008 ***
Informal care from inside the household	5.31	7.86	8.27	10.42	6.78	8.68	0.003 ***
*Age categories*							n.a.
Age 50 to 65	51.38	44.47	28.90	25.79	15.19	13.89	
Age 65 to 80	43.21	46.23	52.88	53.11	58.18	59.72	
Age 80+	5.41	9.31	18.22	21.09	26.63	26.39	
Gender: female	58.02	58.32	57.79	59.72	78.51	69.31	
*Education*							
No education	22.15	21.6	22.44	19.69	21.88	22.84	0.029 **
Low education	64.21	64.69	63.81	65.47	67.53	64.23	0.782
Medium education	6.46	6.65	6.51	7.42	6.12	6.53	0.000 **
High education	7.18	7.06	7.24	7.42	4.47	6.4	0.187
*Marital status*							
Married	84.16	82.22	77.11	74.09	66.32	63.92	0.000 ***
Registered partnership	0.31	0.21	0.24	0.25	0.23	0.22	0.041 **
Separated	1.74	1.65	1.32	1.52	0.70	0.78	0.000 ***
Never married	0.92	1.03	0.84	1.14	0.71	0.79	0.038 **
Divorced	1.33	1.45	1.31	2.03	2.34	2.53	0.042 **
Widowed	11.54	13.44	19.18	20.97	29.91	31.76	0.000 ***
*Current job situation*							
Retired	34.91	40.64	50.36	54.76	49.06	53.27	0.000 ***
Employed or self-employed	19.82	16.75	10.79	8.39	4.44	7.02	0.000 ***
Unemployed	4.09	2.17	2.40	2.80	1.17	2.23	0.008 ***
Permanently sick or disabled	4.10	4.14	3.48	3.30	3.74	4.11	0.062 *
Homemaker	37.08	36.30	32.97	30.75	41.59	33.37	0.000 ***
Household net income (SD)	24,331.03 (26,155.39)	20,617.95 (29,514.6)	20,664.69 (27,475.82)	17,630.19 (12,944.29)	15,238.37 (10,137.66)	16,083.22 (10,872.63)	0.000 ***
Living in a rural area	46.52	48.96	49.18	49.25	54.02	51.42	0.003 **
Number of children (SD)	2.91 (1.52)	2.91 (1.52)	2.90 (1.47)	2.87 (1.45)	2.80 (1.46)	2.83 (1.42)	n.a.
Number of grandchildren (SD)	2.76 (3.27)	3.08 (3.51)	3.56 (3.28)	3.62 (3.39)	3.86 (3.47)	4.12 (3.58)	n.a.
Children living in household	59.35	51.29	43.88	40.79	37.15	35.21	0.000 ***
*Functional limitation level*							
No limitation/Not eligible	97.02	96.16	90.13	90.26	88.38	89.91	0.043 **
Mild limitation	2.36	2.28	5.42	3.46	6.40	3.85	0.000 ***
Moderate limitation	0.41	0.73	2.89	2.95	1.90	2.60	0.000 ***
Severe limitation	0.21	0.83	1.56	3.33	3.32	3.64	0.000 ***
*Self-perceived health*							
Excellent	4.29	3.00	2.41	3.23	2.11	4.21	0.000 ***
Very good	14.50	8.79	10.08	9.40	7.94	16.37	0.000 ***
Good	40.45	40.64	35.17	36.09	36.45	34.69	0.031 **
Fair	30.95	33.82	36.01	35.45	35.28	28.20	0.004 ***
Poor	9.81	13.75	16.33	16.90	18.22	16.39	0.000 ***
Number of chronic conditions (SD)	1.14 (1.16)	1.10 (1.07)	1.39 (1.23)	1.45 (1.29)	1.46 (1.17)	1.99 (1.70)	0.000 ***
Depression	35.51	32.14	36.23	32.58	37.34	39.54	0.902
*Body Mass Index categories*							
Underweight	0.31	0.52	0.84	0.89	0.93	1.17	0.218
Normal weight	25.23	24.20	24.82	24.27	26.87	29.08	0.086 *
Overweight	47.80	47.36	46.76	48.03	46.03	40.75	0.074 *
Obesity	26.66	27.92	27.58	26.81	26.17	18.46	0.182

*** *p* < 0.01, ** *p* < 0.05, * *p* < 0.1. Means are presented as percentages, unless indicated otherwise. n.a. stands for not applicable. ^£^ The question about home care was excluded in the questionnaire of wave 4. Hence, the only measure of formal care available in wave 4 is nursing home care. The results from wave 4 should then be interpreted with caution.

**Table 2:** Results from the GLM regression with Gamma distribution and identity link on household income.

Variables	Coefficients Model 1	Coefficients Model 2	Coefficients Model 3	Coefficients Model 4
*Waves*				
Wave 1 (year 2004)	2686 ***	2857 ***	2275 ***	2298 ***
	**(858.2)**	**(788.6)**	**(751.1)**	**(726.4)**
Wave 4 (year 2010)	769.5	687.8	381.7	345.7
	(656.8)	(633.6)	(550.7)	(563.6)
Wave 5 (year 2013)	−529.4	−448.0	7.909	−41.44
	(631.9)	(575.0)	(565.1)	(557.4)
Wave 6 (year 2015)	−909.5	−888.7	−265.8	−296.5
	(634.7)	(567.6)	(538.8)	(562.5)
Wave 7 (year 2017)	−828.3	−797.3	−228.7	−244.4
	(606.2)	(555.1)	(506.9)	(519.2)
*Dependency level*				
Mild limitation	1755	796.3	1066	2466
	(3647)	(2814)	(3221)	(3262)
Moderate limitation	356.3	1330	−176.0	577.3
	(5225)	(4086)	(3872)	(3833)
Severe limitation	−1671	−2720 *	−3314 ***	−3771 ***
	(1216)	**(1486)**	**(1257)**	**(1436)**
*Wave # dependency*				
Wave 1 # mild limitation	4092	3474	−2185	−3450
	(5659)	(5011)	(4038)	(3998)
Wave 4 # mild limitation	−1037	−598.5	70.64	−1512
	(3894)	(3036)	(3435)	(3473)
Wave 5 # mild limitation	−511.4	772.3	−444.8	−2335
	(3838)	(3206)	(3496)	(3675)
Wave 6 # mild limitation	−2663	−1703	−1315	−2059
	(3680)	(2887)	(3307)	(3407)
Wave 7 # mild limitation	−2588	−1611	−1192	−1927
	(3592)	(2834)	(3187)	(3271)
Wave 1 # moderate limitation	−3414	−5663	−2284	−5376
	(6159)	(5104)	(4935)	(8798)
Wave 4 # moderate limitation	−1844	−3156	−1866	−1972
	(5307)	(4245)	(4024)	(3955)
Wave 5 # moderate limitation	2204	−122.9	−177.4	−3146
	(5660)	(4793)	(4555)	(4499)
Wave 6 # moderate limitation	1774	270.6	2667	1511
	(5345)	(4269)	(4365)	(5499)
Wave 7 # moderate limitation	1798	1909	2485	1908
	(5358)	(4761)	(4719)	(4983)
Wave 1 # severe limitation	1042	917.1	4030	−2693
	(3859)	(3226)	(4466)	(1752)
Wave 4 # severe limitation	39,386	39,066	47,107	75,614
	(35,173)	(35,361)	(45,111)	(71,507)
Wave 5 # severe limitation	2380	2671	2964 *	3668 *
	(1544)	(1726)	**(1465)**	**(1765)**
Wave 6 # severe limitation	3125	5010 *	5045 *	3499 *
	(2526)	**(2925)**	**(2364)**	**(1810)**
Wave 7 # severe limitation	2873	3191	3202	2688
	(2263)	(2309)	(2046)	(1652)
N (Observations)	4364	4364	4364	4364
N (Individuals)	1173	1173	1173	1173
Log−pseudolikehood	−42,886.82	−42,761.83	−40,007.74	−38,736.81

Clustered standard errors at the individual level in parentheses. *** *p* < 0.01, * *p* < 0.1. Reference categories: wave 2 (years 2006/2007), not limited/not eligible for receiving benefits from the Dependency Act, age 50 to 65, male, no education, married, retired, excellent self-perceived health status, and with normal weight. In Model 1, wave dummies, the different categories of functional limitation level, and the interaction (denoted by #) between those two categorical variables, as well as age, gender, education, and marital status were included. Model 2 additionally adjusted for employment status. Model 3 living conditions, such as living in a rural area or the number of children and grandchildren. Health status variables (self-perceived health status, number of chronic conditions, depression, and Body Mass Index categories) are introduced in a fourth regression model. Significant results are in bold.

**Table 3:** Results from the logit regressions with random effects on formal care use.

Variables	Odds Ratio Model 1	Odds Ratio Model 2	Odds Ratio Model 3	Odds Ratio Model 4
*Waves*				
Wave 1 (year 2004)	1.310	1.357	1.459 *	1.593 **
	(0.272)	(0.284)	**(0.306)**	**(0.343)**
Wave 4 (year 2010)	0.0766 ***	0.0766 ***	0.0310 ***	0.00875 ***
	**(0.0317)**	**(0.0323)**	**(0.0192)**	**(0.00959)**
Wave 5 (year 2013)	0.902	0.928	0.834	0.755
	(0.186)	(0.193)	(0.184)	(0.180)
Wave 6 (year 2015)	1.031	1.053	1.115	0.985
	(0.241)	(0.251)	(0.272)	(0.270)
Wave 7 (year 2017)	1.026	1.058	1.097	0.979
	(0.237)	(0.250)	(0.269)	(0.266)
*Dependency level*				
Mild limitation	2.515 *	2.176	1.915	0.616
	**(1.322)**	(1.167)	(1.087)	(0.485)
Moderate limitation	7.108 ***	5.571 ***	9.112 ***	9.060 ***
	**(4.600)**	**(3.233)**	**(4.516)**	**(6.226)**
Severe limitation	4.016 **	2.808	3.565	4.421
	**(2.744)**	(1.950)	(2.853)	(4.665)
*Household income*				
Low household income	0.932	0.888	0.871	0.766
	(0.179)	(0.177)	(0.179)	(0.165)
Medium household income	0.943	0.936	0.902	0.919
	(0.264)	(0.263)	(0.265)	(0.280)
*Dependency # income*				
Mild limitation # low household income	0.765	0.815	0.897	1.179
	(0.484)	(0.530)	(0.611)	(1.074)
Mild limitation # high household income	0.694	0.897	1.897	4.097
	(0.764)	(0.988)	(2.399)	(5.934)
Moderate limitation # low household income	0.603	0.679	0.377	0.252
	(0.512)	(0.537)	(0.305)	(0.246)
Moderate limitation # high household income	4.331	5.815	4.769	3.057
	(6.526)	(8.676)	(6.825)	(4.267)
Severe limitation # low household income	1.010	1.147	0.978	0.417
	(0.781)	(0.881)	(0.891)	(0.501)
Severe limitation # high household income	-	-	-	-
			
N (Observations)	4364	4364	4364	4364
N (Individuals)	1173	1173	1173	1173
Log-pseudolikehood	−712.37	−700.52	−681.88	−617.91
Prob > chi^2^	0.000 ***	0.000 ***	0.000 ***	0.000 ***

Clustered standard errors at the individual level in parentheses. *** *p* < 0.01, ** *p* < 0.05, * *p* < 0.1. Reference categories: wave 2 (years 2006/2007), not limited/not eligible for receiving benefits from the Dependency Act, medium household income, age 50 to 65, male, no education, married, retired, excellent self-perceived health status, and with normal weight. In Model 1, wave dummies, the different categories of functional limitation and income levels, and the interaction (denoted by #) between those two latter categorical variables, as well as age, gender, education, and marital status were included. Model 2 additionally adjusted for employment status. Model 3 adds living conditions, such as living in a rural area or the number of children and grandchildren. Health status variables (self-perceived health status, number of chronic conditions, depression, and Body Mass Index categories) are introduced in a fourth regression model. Significant results are in bold.

**Table 4:** Results from the logit regressions with random effects on informal care use.

Variables	Odds Ratio Model 1	Odds Ratio Model 2	Odds Ratio Model 3	Odds Ratio Model 4
*Waves*				
Wave 1 (year 2004)	0.912	0.917	0.948	0.998
	(0.112)	(0.112)	(0.122)	(0.139)
Wave 4 (year 2010)	0.864	0.863	0.862	0.808
	(0.112)	(0.112)	(0.115)	(0.115)
Wave 5 (year 2013)	0.944	0.931	0.978	0.951
	(0.125)	(0.124)	(0.136)	(0.141)
Wave 6 (year 2015)	0.550 ***	0.545 ***	0.534 ***	0.554 ***
	**(0.0941)**	**(0.0936)**	**(0.0966)**	**(0.103)**
Wave 7 (year 2017)	0.531 ***	0.522 **	0.507 **	0.489 **
	**(0.089)**	**(0.091)**	**(0.102)**	**(0.116)**
*Dependency level*				
Mild limitation	8.846 ***	8.510 ***	8.209 ***	5.177 ***
	**(3.120)**	**(3.059)**	**(3.061)**	**(1.950)**
Moderate limitation	75.05 ***	69.80 ***	68.60 ***	72.37 ***
	**(68.87)**	**(66.86)**	**(64.80)**	**(50.34)**
Severe limitation	28.54 ***	26.48 ***	20.91 ***	11.95 ***
	**(22.26)**	**(20.51)**	**(16.53)**	**(11.41)**
*Household income*				
Low household income	1.263 **	1.255 **	1.166	1.073
	**(0.145)**	**(0.143)**	(0.138)	(0.133)
Medium household income	1.148	1.180	1.300	1.313
	(0.185)	(0.192)	(0.222)	(0.227)
*Dependency # income*				
Mild limitation # low household income	0.536	0.546	0.617	0.670
	(0.233)	(0.240)	(0.282)	(0.316)
Mild limitation # high household income	-	-	-	-
			
Moderate limitation # low household income	0.109 **	0.108 *	0.135 *	0.0827 **
	**(0.121)**	**(0.123)**	**(0.149)**	**(0.0811)**
Moderate limitation # high household income	1.676	1.676	1.421	1.323
	(1.215)	(1.219)	(1.211)	(1.143)
Severe limitation # low household income	0.352	0.364	0.468	0.344
	(0.313)	(0.321)	(0.427)	(0.379)
Severe limitation # high household income	0.0889	0.0835	0.104	-
	(0.143)	(0.143)	(0.182)	
N (Observations)	4364	4364	4364	4364
N (Individuals)	1173	1173	1173	1173
Log-pseudolikehood	−1636.76	−1629.82	−1563.51	−1423.85
Prob > chi^2^	0.000 ***	0.000 ***	0.000 ***	0.000 ***

Clustered standard errors at the individual level in parentheses. *** *p* < 0.01, ** *p* < 0.05, * *p* < 0.1. Reference categories: wave 2 (years 2006/2007), not limited/not eligible for receiving benefits from the Dependency Act, medium household income, age 50 to 65, male, no education, married, retired, excellent self-perceived health status, and with normal weight. In Model 1, wave dummies, the different categories of functional limitation and income levels, and the interaction (denoted by #) between those two latter categorical variables, as well as age, gender, education, and marital status were included. Model 2 additionally adjusted for employment status. Model 3 adds living conditions, such as living in a rural area or the number of children and grandchildren. Health status variables (self-perceived health status, number of chronic conditions, depression, and Body Mass Index categories) are introduced in a fourth regression model. Significant results are in bold.

## Data Availability

This paper uses data from SHARE waves 1, 2, 4, 5, and 6 (DOIs 10.6103/SHARE.w1.600, 10.6103/ SHARE.w2.600, 10.6103/SHARE.w4.600, 10.6103/SHARE. w5.600, and 10.6103/SHARE.w6.600). The SHARE data collection was primarily funded by the European Commission through FP5 (QLK6-CT-2001-00360), FP6 (SHARE-I3, RII-CT-2006-062193; COMPARE, CIT5-CT-2005-028857; and SHARELIFE, CIT4-CT-2006-028812), and FP7 (SHARE-PREP, 211909; SHARE-LEAP, 227822; and SHARE M4, 261982). Additional funding from the German Ministry of Education and Research, the Max Planck Society for the Advancement of Science, the US National Institute on Ageing (U01_AG09740-13S2, P01_AG005842, P01_AG08291, P30_AG12815, R21_AG025169, Y1-AG-4553-01, IAG_BSR06-11, OGHA_04-064, and HHSN271201300071C), and various national funding sources is gratefully acknowledged (see www.share-project.org (accessed on 24 April 2020)).

## Supplementary Materials

The following are available online at https://www.mdpi.com/article/10.3390/ijerph18084339/s1, Table S1: Comparison of Rating Scale of Dependency Act and information from the Survey of Health, Ageing and Retirement in Europe (SHARE).

## Abbreviations

ADL	Activities of Daily Living
DA	Dependency Act
GDP	Gross Domestic Product
GLM	Generalized Linear Model
IADL	Instrumental Activities of Daily Living
ISCED	International Standard Classification of Education
LPM	Linear Probability Model
LTC	Long-Term Care
OECD	Organisation for Economic Co-Operation and Development
OLS	Ordinary Least Square
OR	Odds Ratio
SAAD	System for Promotion of Personal Autonomy and Assistance for Persons in a Situation of Dependency
SHARE	Survey of Health, Ageing and Retirement in Europe
