# Supplementary material for: Dependent, Poorer, and More Care-Demanding? An Analysis of the Relationship between Being Dependent, Household Income, and Formal and Informal Care Use in Spain
Source: Int J Environ Res Public Health. 2021 Apr 19;18(8):4339. doi: 10.3390/ijerph18084339 (PMC8073092; doi:10.3390/ijerph18084339)
Supplement: Supplementary file 1 [file ijerph-18-04339-s001.zip › ijerph-1143756-supplementary.pdf]

Table S1. Comparison of Rating Scale of Dependency Act and information from the Survey of Health, Ageing and Retirement in Europe (SHARE).

Rating Scale of Dependency Act			SHARE		
**1.**	**Eating and drinking**	16.8	**1.**	**Eating and drinking**	16.8
	-Recognize and / or reach the food served	0.25		Eating and drinking tasks	1
	-Cut food into pieces	0.20			
	-Use folk and knife to bring	0.30			
	-The beverage container to the mouth	0.25			
**2.**	**Personal hygiene related to urination / defecation**	14.8	**2.**	**Personal hygiene related to urination / defecation**	14.8
	-Go to appropiate place	0.20		- Using the toilet, including getting up or down	1
	-Manage clothing				
	-Move properly	0.15			
	-Move properly	0.30			
	- Clean yourself				
		0.35			
**3.**	**Bathing**	8.8	**3.**	**Bathing**	8.8 + 2.9[1]
	-Open and close the tap	0.15		Bathing or showering	1
	-Washing the hands	0.20			
	-Go to the bath	0.15			
	-Wash the upper part of the body	0.25			
	-Wash the down part of the body	0.25			
**4.**	**Doing other corporal tasks**	2.9	**4.**	**Doing other corporal tasks**	0
	-Comb	0.30		Not available in our data. We imputed the weight of "doing other corporal tasks" to "bathing"	
	-Cut the nails	0.15			
	-Wash the hair	0.25			
	-Wash the teeth				
**5.**	**Dressing**	11.9	**5.**	**Dressing**	11.9
	-Manage the property clothing	0.15		Dressing and undressing, including shoes and shocks	1
	-Put the shoes	0.10			
	-Buttoning	0.15			
	-Dressing the clothing in the down part of the body	0.30			
	-Dressing the clothing in the upper part of the body	0.30			
**6.**	**Health**	2.9	**6.**	**Health**	2.9

[1] Since limitations in doing other corporal tasks is not available in SHARE, its weight was imputed to "bathing".

Rating Scale of Dependency Act			SHARE		
	-Ask for therapeutic assistence	0.15		-Taking medications	0.55
	-Applied the tereupertic recommendations	0.10		-Use the telephone	0.5
	-Avoid risks into home	0.25			
	-Avoid riks out of home	0.25			
	-Ask for help in case of urgence	0.25			
**7.**	**Change and move the possition**	9.4	**7.**	**Change and move the possition**	9.4
	-Change the body from laid to sit down in bed	0.10		- Getting in or out of bed	1
	-Change the body from sit down in stand up	0.10			
	-Change the body from stand up in sit down in a chair	0.10			
	-Transfer while sit down	0.10			
	-Transfer while laid	0.10			
	-Chang the gravitatory point while sit down	0.20			
	-Sit down	0.15			
	-Stand up	0.15			
**8.**	**Move into home**	12.3	**8.**	**Move into home**	12.3
	-Move to dress	0.25		-Walking across a room	1
	-Move to eat	0.15			
	-Move to wash	0.10			
	-Move not related to self-care	0.25			
	-Move thing in the room	0.10			
	-Move around the romms	0.15			
**9.**	**Move out of home**	12.2	**9.**	**Move out of home**	12.2
	-Move out of building	0.25		- Leaving the house independently / accessing transportation	1
	-Move around of building	0.25			
	-Move shortly in known-enviroment	0.20			
	-Move shortly in unknown-enviroment	0.15			
	-Move far away in known-enviroment	0.10			
	-Move far away in unknown-enviroment	0.05			
**10.**	**Housemake tasks**	8	**10.**	**Housemake tasks**	8
	-Prepare meal	0.45		-Prepare meal	0.45
	-Do the shopping	0.25		-Shopping for groceries	0.25
	-Clean the house	0.20		-Doing work around the house or garden	0.20
	-Wash clothing	0.10		- Doing personal laundry	0.10

Source: adapted from Oliva-Moreno et al. (2015) and by our own using SHARE and la Resolución de 29 de junio de 2010 por la que se publica el Acuerdo del Consejo Territorial del Sistema para la Autonomía y

Atención a la Dependencia sobre modificación del baremo de valoración de la situación de dependencia establecido en el Real Decreto 504/2007, de 20 de abril.
